# A prospective comparison of short term results and functional recovery after laparoscopic subtotal colectomy and antiperistaltic cecorectal anastomosis with short colonic reservoir vs. long colonic reservoir

**DOI:** 10.1186/s12876-015-0257-7

**Published:** 2015-03-18

**Authors:** Dong Wei, Jian Cai, Yang Yang, Ting Zhao, Hui Zhang, Changshan Zhang, Yuanyao Zhang, Jianfeng Zhang, Fengbo Cai

**Affiliations:** 1 Institute of Anal-Colorectal Surgery, No. 150 Central Hospital of PLA, Luoyang, 471031 China; 2 The Second Military Medical University, Shanghai, China

**Keywords:** Slow Transit Constipation (STC), Laparoscopic subtotal colectomy, Antiperistaltic cecorectal anastomosis, Quality of life

## Abstract

**Background:**

To observe and compare the short term results and functional recovery of laparoscopic subtotal colectomy with antiperistaltic cecorectal anastomosis (LSCACRA) in the treatment of Adult slow transit constipation (STC) with two different reservoir length: short colonic reservoir and long colonic reservoir.

**Methods:**

All STC patients treated with LSCACRA between April 2007 and December 2011 at our institution were followed up. Patients with 2 cm to 3 cm ascending colon preserved above the ileocecal junction were designated as observation group, whereas those preserved by 10 cm to 15 cm were classified as control group. 41 cases in the observation group and 40 cases in the control group were enrolled. Preoperative and outcome parameters of patients were collected, including gender, age, body mass index, operative time , blood loss, first flatus time, hospital stay, postoperative complications, Wexner constipation scale(WCS), Wexner incontinence scale, gastrointestinal quality of life index(GIQLI), abdominal pain intensity scale(APIS), abdominal pain frequency scale(APFS) and abdominal bloating scale(ABS).

**Results:**

Laparoscopic surgeries were successfully carried out for all patients, without any case transferred to laparotomy or death related to surgery. The operative time, blood loss, first flatus time, and days of hospital stay of the two groups did not show significant differences. We found no significant differences on complications (Clavien–Dindo grade > I) between the two groups. No patient exhibited anastomotic leak. No fecal incontinence occurred in both groups. On the 3^rd^, 6^th^ and 12^th^ month after operation, the parameters of both groups significantly improved compared with the preoperative conditions (P < 0.05) except the APIS at 3^rd^ and 6^th^ month in control group. On the 3^rd^, 6^th^ and 12^th^ month after operation, the Functional Recovery outcomes of WCS、GIQLI、APIS、APFS and ABS in the observation group were superior to those in the control group (P < 0.05).

**Conclusion:**

LSCACRA has a significant effect in the treatment of STC in adult. Postoperative outcomes can be optimized by shortening the length of the preserved ascending colon above the ileocecal junction, which promise better life quality of patients. Trial registration: Chinese Clinical Trial Registry ChiCTR-OPC-14005280, 2014-09-29.

**Electronic supplementary material:**

The online version of this article (doi:10.1186/s12876-015-0257-7) contains supplementary material, which is available to authorized users.

## Background

Constipation is a common problem with 16% of women and 12% of men met the symptom criteria [1], which severely affects the life quality of patients. The surgical approach is currently the only way to treat long-term intractable slow transit constipation (STC) that is not responsive to pharmacological therapy. Surgical treatment for STC has two main approaches. One method is total colectomy with ileorectal anastomosis (TC-IRA), which is the most widely adopted procedure with a high cure rate for STC [2-4]. Although constipation is relieved by increasing the frequency of bowel movement in the majority of patients after TC-IRA, some symptoms such as bloating, abdominal pain, intractable diarrhea, loss of nutrient substance and ileus are still common problems [4-7]. Given the risks, surgeons are very careful with this choice.

The other approach is subtotal colectomy with cecorectal anastomosis (SCCRA), which is conducive to the absorption of aqueous electrolyte, bile salts, and vitamins; alleviation of severe postoperative diarrhea; and remarkably low incidence of postoperative ileus. These effects improve the life quality of patients significantly [8-13]. SCCRA can be divided into 4 main methods:isoperistaltic cecorectal anastomosis, isoperistaltic anastomosis by cecum translational rotation in the left iliac fossa, side-to-end cecorectal anastomosis and antiperistaltic cecorectal anastomosis [8,14-18]. The former three methods need a certain degree rotation of bowel or mesentery, but it’s will result in increasing the possibility of bowel obstruction and blood circulation barriers. While the antiperistaltic cecorectal anastomosis don’t need to change the position of ileocecal junction and mesentery. In 2001, Sarli [8] first reported the treatment of constipation using subtotal colectomy with antiperistaltic cecorectal anastomosis (SCACRA). Since then, scholars have conducted related researches [9,19-24].

The efficacy of SCACRA can be evaluated based on a few existing retrospective studies, but without unified evaluation criteria. First, the inclusion criteria of different studies are not unified. Jiang [19] completely excluded patients with outlet obstruction constipation and pelvic floor dysfunctions. By contrast, Sarli, Marchesi, and others [8,9,19,20,24] included patients with outlet obstruction constipation as a minor symptom. Second, research methods and parameters among different institutions or one institution at different times showed differences. In the early studies of Sarli et al. [8,19,23,24], Wexner incontinence scale (WIS) [25], Wexner constipation scale (WCS) [26], gastro-intestinal quality of life index (GIQLI) [27], and other objective evaluation parameters were not widely adopted. They mainly used the subjective efficacy of patients as an evaluation criterion. Marchesi [9,20] and Jiang [21] added the aforementioned parameters of objective evaluation, but did not study the preoperative values of these parameters. In their research, abdominal pain directly stemmed from GIQLI. The degree and frequency of abdominal pain were insufficiently recorded. The number of cases was also relatively small.

At the same time, several proposals have been forwarded regarding the preserved length of the ascending colon above the ileocecal junction. The four main resection position above the ileocecal junction are as follows: several centimeters [22], 5-7 cm [21], maximum length 8 cm [28], 10 cm [20] and 10-15 cm [8,19]. However, which proposal is better has not been studied to date. In this study we compared two different resectional length: 10–15 cm above the ileocecal junction and 2-3 cm above the ileocecal junction.

## Methods

### Patients

In our institution, laparoscopic SCACRA (LSCACRA) has gone through two stages. Between April 2007 and August 2009, 10 cm to 15 cm of the ascending colon above the ileocecal junction was reserved, and all these 42 patients were taken as the control group. Since September 2009 to present, resection was made 2 cm to 3 cm above the ileocecal junction, and the patients consecutively treated were taken as the observation group (42 patients). The observation group had one case lost to follow up, whereas the control group had two cases lost to follow up. Finally, 41 cases in the observation group and 40 cases in the control group were enrolled. Preoperative examination contains gastrointestinal transit time (GITT) studies, defecography, anorectal manometry, barium enema, colonoscopy and routine preoperative examinations of colon resection.

In this study, the selection of surgical indications are as follows. Inclusion criteria: 1. the Rome III diagnosis criteria for constipation; 2. Diagnosis of STC (positive results with at least twice GITT studies before operation); 3. Chronic (non-surgical treatment for more than 5 years), severe (WCS > 15), Refractory (long-term dependence of large doses of laxatives or enemas) STC; 4. The exclusion of other colorectal organic disease; 5. Age:18-70years. Exclusion criteria: 1. American Society of Anesthesiologists (ASA) scores >3; 2. Abnormal liver function and kidney function; 3. Patients with psychiatric symptoms or a history of Psychiatric disease; 5.patients showing obvious symptoms of outlet obstruction (such as higher bowel movement frequency, difficulty in discharging non-dry stool , anal and rectal disfunction, etc.); 6. Patients with a history of abdominal big surgery; 7. Patients with life threatening diseases such as cancer.

### Surgical procedure

**Figure 1:**
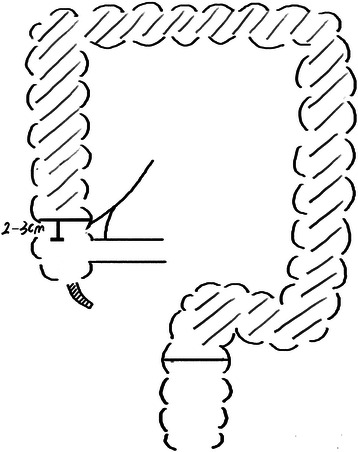
**Resection area of observation group.** The shadow is resection area.

**Figure 2:**
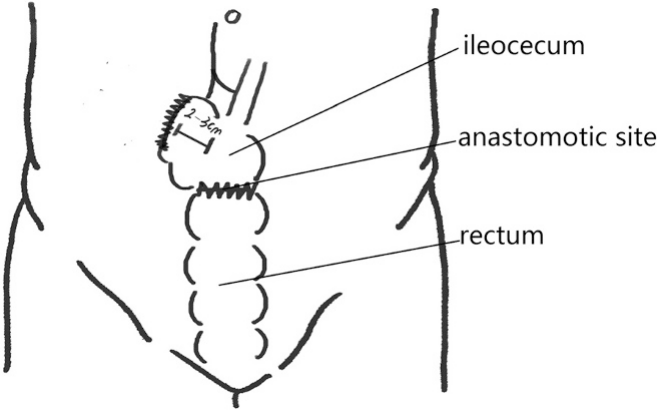
**Post-operational schematic diagram of observation group.**

**Figure 3:**
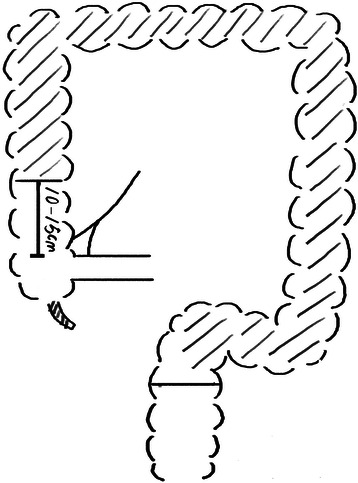
**Resection area of control group.** The shadow is resection area.

**Figure 4:**
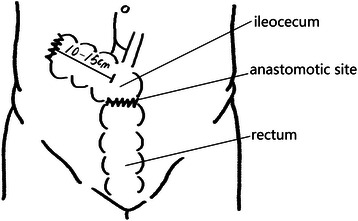
**Post-operational schematic diagram of control group.**

All operations were performed by Dr. Wei Dong. We performed LSCACRA through a five-trocar approach with the same surgical procedure in both groups, except for the length of the preserved ascending colon above the ileocecal junction. We preserved 10 cm to 15 cm or 2 cm to 3 cm of the colon from the upper edge of the ileocecal valve along the mesentery of the ascending colon. Referring to Figures 1, 2, 3 and 4.

Pneumoperitoneum was established by a Veress needle at an intra-abdominal pressure of 12 mmHg. The five trocars were distributed in the following way. A 10 mm trocar for the camera was placed 2 cm to 3 cm below the umbilicus for the 30° camera. Two trocars were placed at the outer edge of right rectus abdominis, forming a regular triangle with side length of about 10 cm from the umbilicus. The trocar at the upper abdomen was 5 mm and the one at the lower was 12 mm. The rest two trocars were placed in the same way at the outer edge of left rectus abdominis, but the trocar at the upper was 12 mm and the one at the lower was 5 mm.

The surgeon first stood on the right side of the patient, mobilizing the left transverse colon, splenic flexure, descending colon, sigmoid colon and upper rectum. The omentum was protected carefully. The 45-mm linear stapler was used to transect the upper rectum. Next, the surgeon moved to the left side of the patient, mobilizing the right transverse colon, hepatic flexure of colon, ascending colon and ileocecal junction. The omentum and end vessels of ileum were protected carefully. A McBurney incision is then performed to take out and resect the colon. The linear stapler was used to transect the ascending colon (with the upper edge of ileocecal valve as the baseline, we calculated the preserved length along the mesocolon of ascending colon upwards). Then Appendectomy is performed. The head of a 33 mm circular stapler is introduced into the cecum through an incision at the bottom of cecum and secured with a purse-string suture. The stapler is introduced into rectal stump and the antiperistaltic cecorectal anastomosis is created. The drainage tube was placed at pouch of Douglas, and the abdominal cavity was closed.

### Patient data acquisition

We collected the general information of patients (gender, age and body mass index), operative parameters (operative time and blood loss) and postoperative recovery (first flatus time, hospital stay and postoperative complications). We evaluated functional recovery by collecting the data of bowel movement (BM), WCS, WIS, GIQLI , abdominal bloating scale (ABS), abdominal pain intensity scale(APIS) and abdominal pain frequency scale(APFS) before and every 3, 6, and 12 months after the operation. WCS is a validated and internationally adopted questionnaire used to quantify the constipation grade of a patient, which ranges from 0 (best) to 30 (worst). WIS is a validated and internationally adopted questionnaire that has five items to quantify incontinence grade, frequency, and its effect on ordinary life. Each question is answered on a scale of 0 to 4, and the global score ranges from 0 (best) to 20 (worst). GIQLI ranging from 0 (worst) to 144(best) is a validated quality-of-life questionnaire consisting of 36 questions designed to evaluate specific gastrointestinal symptoms and the effect of the disease on the physical, psychological, and social aspects of patients. Each question consists of 5 levels from 0 to 4(0 = all the time; 1 = often; 2 = sometimes; 3 = occasionally; 4 = never). The abdominal bloating score (ABS) and abdominal pain frequency scale (APFS) were both deducted from the questions of GIQLI. They consist of 5 levels from 0 to 4 (0 = never; 1 = occasionally; 2 = sometimes; 3 = often; 4 = all the time). But the order reverse to GIQLI. The abdominal pain intensity was indicated by the numerical rating scale (0–10) [29]. We defined patients with APFS greater than 1 as existing abdominal pain associated with surgery and patients with APFS greater than 2 as existing frequent abdominal pain. We defined patients with ABS greater than 1 as existing abdominal bloating associated with surgery and patients with ABS greater than 2 as existing frequent abdominal bloating. Operative complications were graded using the Clavien–Dindo score tool [30]. We noted whether complications with at least level II were taken as research indicators. All the post-operation data were collected through questionnaire by clinical visit or phone. This study began in September 2009. Partial data of patients in control group come from historical information.

### Comparative method and statistical treatment

We compared the postoperative parameters at 3, 6, and 12 months after the operation of the two groups, including WCS, APIS, APFS, ABS, GIQLI and BM with preoperative parameters. We studied the variations in parameters among patients in each group. Thus, the effects of LSCACRA in the treatment of STC patients were evaluated. We compared the postoperative parameters at 3, 6, and 12 months after the operation including WCS, APIS, APFS, ABS, GIQLI and BM between the two groups. Thus, the effects of two different surgical methods were evaluated.

The variables were expressed as mean ± standard deviation (SD). T-test of paired data was used for comparison at different time points within each group. For comparison baseline data pre-operation and WIS post-operation between groups, t-test of two independent samples and Pearson chi-square test were applied. For comparison functional recovery post-operation (WCS, APIS, APFS, ABS, GIQLI and BM) between groups, analysis of covariance were applied. P < 0.05 was considered statistically significant. All data were analyzed by PSAW18.

### Ethical statement

The protocol of this study was approved by the Ethics Committees of No. 150 Central Hospital of PLA, and written informed consents were obtained from all patients. The present work conformed to the provisions of the Declaration of Helsinki.

## Results

### Basic information and preoperative data

The basic characteristics of the two groups of patients were similar without significant differences. Both groups mainly comprised females with age range from 22 to 70. The average age of observation group is 50.2; that of control group is 49.2. All patients suffered from severe preoperative constipation. Preoperative WCS of the observation group and the control group were 16.61 and 16.55, respectively. Before the operation, the life quality of patients in the two groups was highly unsatisfactory, which was demonstrated by the extremely low pre-operative GIQLI score (64.61 for the observation group and 63.08 for the control group). The two groups of patients suffered from a certain intensity of abdominal pain and abdominal bloating before the operation. Fecal incontinence never occurred before the operation. Details can be found in Table 1.

**Table 1:** **Clinical characteristics of patients (mean ± SD)**

			Observation group N = 41	Control group N = 40	*P*value
**Basic information**	Gender	M/F	10/31	8/32	0.64
	Age	year	50.24 ± 12.85	49.23 ± 13.42	0.73
	BMI	Kg/ m2	22.80 ± 4.18	21.59 ± 3.52	0.16
**Preoperative data**	WCS	(0–30)	16.61 ± 1.55	16.55 ± 1.30	0.85
	GIQLI	(0–144)	64.61 ± 3.97	63.08 ± 4.02	0.09
	APIS	(0–10)	3.10 ± .97	3.00 ± 1.30	0.70
	APFS	(0–4)	2.39 ± 0.49	2.30 ± 0.46	0.40
	ABS	(0–4)	2.41 ± 0.63	2.28 ± 0.51	0.28
	BM	times/week	2.15 ± 2.42	1.85 ± 1.56	0.52
**Operative and postoperative data**	Operation time	min	210.54 ± 53.45	196.15 ± 57.42	0.25
	Blood lose	ml	141.71 ± 62.73	158.75 ± 63.09	0.23
	Hospital stay	days	13.02 ± 2.08	13.05 ± 2.14	0.96
	Time to first flatus	days	4.37 ± 2.36	4.60 ± 1.58	0.60
	Morbidity (Dindo > I)		4	5	1.00★

Note: BMI: body mass index; WCS: Wexner constipation scale; GIQLI: Gastro-Intestinal Quality of Life Index; APIS: abdominal pain intensity scale; APFS: abdominal pain frequency scale; ABS: abdominal bloating scale; BM: number of bowel movements; WIS: Wexner incontinence scale; Dindo: Clavien-Dindo grade.

★Fisher’s Exact Test.

### Surgical data and postoperative outcomes

Laparoscopic surgeries were successfully carried out for all patients, without any case converted to laparotomy during the operation nor the death related to surgery. The operative time, blood loss, first flatus time, and days of hospital stay of the two groups did not show significant differences. We collected all complications with Clavien–Dindo grade > I, and found no significant differences between the two groups. Details can be found in Table 1. In the observation group, we observed three cases of incomplete intestinal obstruction and two cases of pulmonary infection. By contrast, in the control group were found two cases of incomplete intestinal obstruction and two cases of pulmonary infection. All the 9 cases were healed by conservative treatment. No patient exhibited anastomotic leak. Fecal incontinence did not occur among the patients of both groups when observed 3 and 6 months after the operation. The WIS scores of the observation group and the control group at the 3rd month after the operation were 5.59 ± 2.01 and 5.13 ± 1.92 respectively, presenting no significant difference (P = 0.30). The values at the 6th month were 2.05 ± 1.09 and 2.63 ± 1.50 respectively, also presenting no significant difference (P = 0.05).

We surveyed postoperative BM per day. The findings in the observation and control groups at the 3^rd^ month after the operation were 4.73 ± 2.24 vs. 4.42 ± 2.45(*P* = 0.56), and 2.54 ± 1.58 vs. 2.59 ± 2.21(*P* = 0.97) at the 6^th^ month. The BM of the two groups at the 6^th^ month since the operation was close to that of healthy people.

### Functional recovery

**Figure 5:**
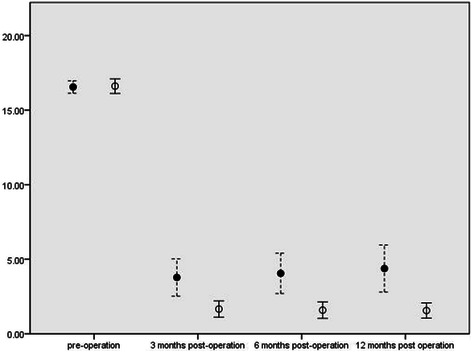
**WCS Scores.** X-axis: scores of WCS; Y-axis: time points pre- and post-operation. ●: mean of WCS in control group;○: mean of WCS in observation group;┊: 95% Confidence interval of WCS in control group;│: 95% Confidence interval of WCS in observation group.

**Figure 6:**
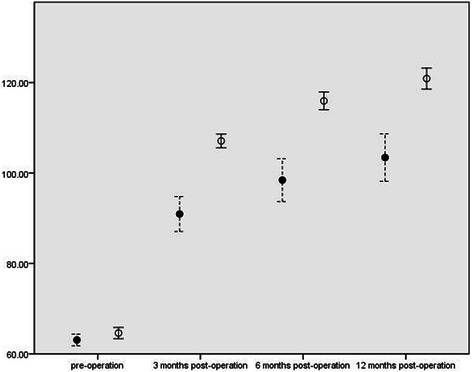
**GIQLI Scores.** X-axis: scores of GIQLI; Y-axis: time points pre- and post-operation. ●: mean of GIQLI in control group;○: mean of GIQLI in observation group; ┊: 95% Confidence interval of GIQLI in control group;│: 95% Confidence interval of GIQLI in observation group.

**Figure 7:**
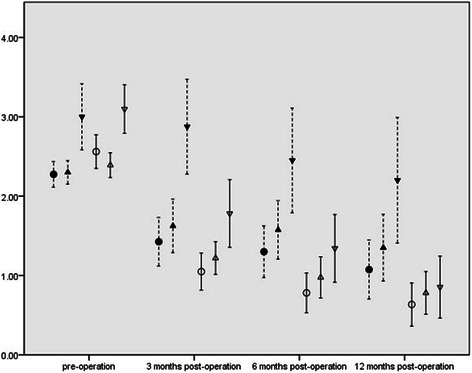
**ABS, APIS and APFS Scores.** X-axis: scores of ABS, APIS and APFS; Y-axis: time points pre- and post-operation. ●: mean of ABS in control group;○: mean of ABS in observation group; ▲: mean of APFS in control group;△: mean of APFS in observation group; ▼: mean of APIS in control group;▽: mean of APIS in observation group; ┊: 95% Confidence interval of each index in control group;│: 95% Confidence interval of each index in observation group.

In both groups, WCS, GIQLI, APFS and ABS scores significantly improved (p < 0.01) in the 3rd, 6th, and 12th month after the operation compared with the pre-operative value(shown in Table 2). However, the observation group exhibited significant improvement of WCS, GIQLI, APFS and ABS in comparing with the control group at each time point after the operation (P < 0.01), as shown in Figures 5, 6, 7 and Table 3.

**Table 2:** **Functional recovery results between pre- and post- operation in each group (mean ± SD)**

	Observation group N = 41				Control group N = 40			
	Pre-operation	post-operation		*p*value	Pre-operation	post-operation		*p*value
**WCS**	16.61 ± 1.55	3 months	1.66 ± 1.73	<1 × 10^−6^	16.55 ± 1.30	3 months	3.78 ± 3.91	<1 × 10^−6^
		6 months	1.59 ± 1.76	<1 × 10^−6^		6 months	4.05 ± 4.23	<1 × 10^−6^
		12 months	1.56 ± 1.61	<1 × 10^−6^		12 months	4.38 ± 4.93	<1 × 10^−6^
**GIQLI**	64.61 ± 3.97	3 months	107.09 ± 4.80	<1 × 10^−6^	63.08 ± 4.02	3 months	90.93 ± 12.06	<1 × 10^−6^
		6 months	115.95 ± 6.19	<1 × 10^−6^		6 months	98.43 ± 14.84	<1 × 10^−6^
		12 months	120.88 ± 7.39	<1 × 10^−6^		12 months	103.43 ± 16.42	<1 × 10^−6^
**APIS**	3.10 ± .97	3 months	1.78 ± 1.35	1 × 10^−6^	3.00 ± 1.30	3 months	2.88 ± 1.87	0.606
		6 months	1.34 ± 1.35	<1 × 10^−6^		6 months	2.45 ± 2.06	0.052
		12 months	0.88 ± 1.25	<1 × 10^−6^		12 months	2.20 ± 2.47	0.020
**APFS**	2.39 ± 0.49	3 months	1.22 ± 0.652	<1 × 10^−6^	2.30 ± 0.46	3 months	1.63 ± 1.05	0.001
		6 months	0.98 ± 0.82	<1 × 10^−6^		6 months	1.58 ± 1.15	0.001
		12 months	0.76 ± .83007	<1 × 10^−6^		12 months	1.35 ± 1.31	<4 × 10^−5^
**ABS**	2.41 ± 0.63	3 months	1.05 ± 0.74	<1 × 10^−6^	2.28 ± 0.51	3 months	1.43 ± 0.96	4 × 10^−6^
		6 months	0.78 ± 0.79	<1 × 10^−6^		6 months	1.30 ± 1.02	<1 × 10^−6^

Note: WCS: Wexner constipation scale; GIQLI: Gastro-Intestinal Quality of Life Index; APIS: abdominal pain intensity scale; APFS: abdominal pain frequency scale; ABS: abdominal bloating scale.

**Table 3:** **Post-operation functional recovery comparison between the two groups (mean ± SD)**

	Time		Observation group N = 41	Control group N = 40	*p*value
**WCS**	Pre-operation		16.61 ± 1.55	16.55 ± 1.30	
	post-operation	3 months	1.66 ± 1.73	3.78 ± 3.91	0.002
		6 months	1.59 ± 1.76	4.05 ± 4.23	0.001
		12 months	1.56 ± 1.61	4.38 ± 4.93	0.001
**GIQLI**	Pre-operation		64.61 ± 3.97	63.08 ± 4.02	
	post-operation	3 months	107.09 ± 4.80	90.93 ± 12.06	<1 × 10^−6^
		6 months	115.95 ± 6.19	98.43 ± 14.84	<1 × 10^−6^
		12 months	120.88 ± 7.39	103.43 ± 16.42	<1 × 10^−6^
**APIS**	Pre-operation		3.10 ± .97	3.00 ± 1.30	
	post-operation	3 months	1.78 ± 1.35	2.88 ± 1.87	0.001
		6 months	1.34 ± 1.35	2.45 ± 2.06	0.002
		12 months	0.88 ± 1.25	2.20 ± 2.47	0.001
**APFS**	Pre-operation		2.39 ± 0.49	2.30 ± 0.46	
	Post-operation	3 months	1.22 ± 0.652	1.63 ± 1.05	0.042
		6 months	0.98 ± 0.82	1.58 ± 1.15	0.009
		12 months	0.76 ± 0.83	1.35 ± 1.31	0.013
**ABS**	Pre-operation		2.41 ± 0.63	2.28 ± 0.51	
	Post-operation	3 months	1.05 ± 0.74	1.43 ± 0.96	0.035
		6 months	0.78 ± 0.79	1.30 ± 1.02	0.008
		12 months	0.63 ± 0.86	1.08 ± 1.16	0.025

Note: WCS: Wexner constipation scale; GIQLI: Gastro-Intestinal Quality of Life Index; APIS: abdominal pain intensity scale; APFS: abdominal pain frequency scale; ABS: abdominal bloating scale.

The APIS values of the observation group after 3, 6, and 12 months of the operation showed significant improvement (P < 0.01) (shown in Table 2). For the controls, APIS values in the 3rd and 6th month after the operation did not significantly improve, and the *p* values were 0.606 and 0.052 respectively. Twelve months after the operation, the value of the APIS of the control group became significantly improved (*p* = 0.02) (shown in Table 2). The observation group presented significant improvement of APIS in comparing with the control group (P < 0.01), as shown in Figure 7 and Table 3.

The abdominal pain incidence was 14.7% after 12 months in the observation group, among which 4.9% experienced frequent abdominal pain. By contrast, the abdominal pain incidence in the control group after 12 months was 42.5%, among which 32.5% experienced frequent abdominal pain. The bloating incidence was 14.7% after 12 months in the observation group, among which 4.9% experienced frequent abdominal bloating. By contrast, the bloating incidence in the control group after 12 months was 25%, among which 22.5% experienced frequent abdominal bloating.

## Discussion

SCACRA comprises two eras, namely, open surgery and laparoscopic surgery. Among the 32 cases reported by Sarli, Marchesi, and others [8,9,19,21-24], 17 cases had open surgery and 15 had laparoscopic surgery. In their institution, almost all recent cases received SCCRA with laparoscopic approach [20]. In 2012, Marchesi [20] compared 15 cases of laparoscopic surgery with 15 cases of open surgery. Although the cases of laparoscopic surgery had longer operation time, the postoperative pains and the days of hospital stay were significantly shorter than those of the group with open surgery. Marchesi [20] holds that laparoscopic SCCRA confirmed the very good functional results of the open approach, with significant advantages for postoperative recovery. The minimally invasive approach did not increase procedural morbidity. In our study, nine patients among all 81 patients exhibited complications at Dindo > level I (11.1%), which was close to the result of open operation (13.3%) reported by Marchesi [20]. Therefore, our results confirmed Marchesi’s point of view that “The minimally invasive approach did not increase procedural morbidity” [20].

The average days of hospital stay of the two groups were 13.02 ± 2.08 and 13.05 ± 2.14 d, which were significantly shorter than the 14.5 ± 2.5 d reported by Jiang [21]. We observed that several key parameters, such as WCS, GIQLI, APIS, APFS and ABS, of the two groups at the 12th month after the operation significantly improved compared with their pre-operative values. Both groups have no severe diarrhea and incontinence.

This result verified that with any approach of preserved length, LSCACRA is a suitable and effective alternative in the treatment of STC. Based on comparisons with earlier reports [8,9,19,24] laparoscopic surgery presents advantages, such as efficacy, smaller wounds and faster recovery.

**Figure 8:**
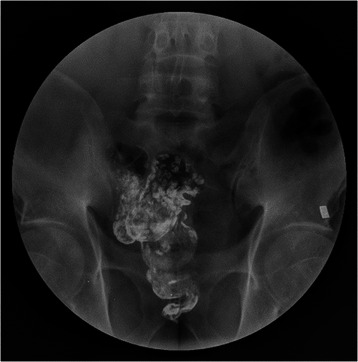
**The barium enema.** Note:large amount of barium has dried in block and remained in cecum and colon. Intestine has been emptied and rectum was basically emptied.

In the beginning, we carried out LSCACRA based on the referred length of 10 cm to 15 cm according to Sarli [8]. Six patients among 42 cases did not present significant improvement in abdominal pain and abdominal bloating after the operation, and even reported subjective feelings of enhanced symptoms. They could not endure the pain and had to take long-term treatment in hospitals. They even requested for re-operations. In the barium enema test, we found cecum expansion in the patients, which was highly similar to what was reported by Marchesi [9]. We also found that these patients had the problem of cecum emptying as shown in Figure 8 (the picture was taken 72 hours after barium enema). The residual cecum and colon were shaped as a blind loop-pouch in which the feces moved inversely. Partial feces stayed for a long time in the reservoir which was hard to be emptied. We assumed that the abdominal pain and abdominal bloat were caused by the blind loop-pouch after operation. The inner pressure in the reservoir may cause abdominal bloating. The large pressure in the cecum leads to reinforced or even spasmodic contraction when the contents of the small intestine enter the colon. Given the excessive length of the blind loop, more intense and longer lasting contractions of the colon will be needed to empty residuals in the blind loop, which worsens colon spasms. The bigger the reservoir was, the more serious the illness would become. We referred to the paper of Jiang [21] in 2008 and found that the difference in the operation technique between Jiang and Sarli was a shorter preserved length of ascending colon from 10 cm to 15 cm to 5 cm to 7 cm. The incidence rate of postoperative abdominal pain was significantly lower than that reported by Marchesi and Sarli. According to Marchesi and Sarli [9], 11 (64.7%) patients had postoperative abdominal pain, among which two patients (11.8%) exhibited frequent abdominal pain. In the research of Jiang [21], although the incidence rates of abdominal pain and bloating were high (17.1% of abdominal pain, 23.5% of abdominal bloating, and 11.8% of postoperative ileus), we observed a significant decline. This result indicates that the shortened length of the colonic reservoir greatly improved the postoperative outcomes and reduced the incidence of postoperative abdominal pain.

Based on the theoretical analysis of subtotal colectomy with antiperistaltic cecorectal anastomosis, we concluded that the surgery is significant in retaining ileocecal valve and preventing rotation of blood vessel and intestine in isoperistaltic anastomosis. It is not necessary to keep longer ascending colon and cecum for the function of pouch. In the literature review, there is no final conclusion about what length of ascending colon and cecum should be retained, so theoretically it is feasible to conduct cecum shortening which also observes the surgery principle. Therefore, we at last performed the surgery of cecum shortening for these 6 patients. The purpose of the new surgery was to solve the problems of feces retention and pneumatosis in the pouch, or minimize even eliminate the pouch. We decided to shorten cecum as much as possible and at the same time guarantee ileocecal valve and original anastomotic stoma intact. This is the reason why we initially fixed 2-3 cm area above the upper edge of ileocecal valve (guaranteeing well function of ileocecal valve and minimized cecum). The patients recovered very well after the second operation and their illness were obviously relieved in three-month after operation. (Details were shown in Additional file 1) Thus, since September 2009 we changed the surgical method to preserving 2-3 cm ascending colon above the ileocecal junction.

The two groups were identical in terms of surgical methods except for the length of colonic reservoir. All operations were performed by the same doctor. Preoperative information of patients showed no significant differences through statistical analysis. The comparison of surgical and postoperative parameters of the two groups could sufficiently interpret the effect of preserved length on postoperative outcomes.

Surgical and postoperative parameters of patients did not show significant difference through statistical analysis. However, we noticed during the comparison of the variations in different parameters in two groups at each postoperative time point that the observation group showed continuous improvement in WCS, GIQLI, APIS, APFS, and ABS at 3, 6, and 12 months after the operation. At 12 months after the operation, the WCS score decreased to 1.56 ± 1.61 and was even better than the WCS score of the general population (2.1 to 3.4) [26]. All patients had WCS score < 8 at 12 months after the operation. GIQLI value was 121.23 at 12 months after the operation, which was close to the level of healthy people (average healthy value is 125.8 ± 13) [27]. Compared with the preoperative conditions, all the values of WCS, GIQLI, APFS, and ABS improved significantly (P < 0.01) in the control group at the 3rd, 6th, and 12th month after operation. APIS was greatly relieved only at the 12th month after the operation (P < 0.05). However, at different postoperative time points, the control group showed remarkable variation in parameters. The WCS score at the 3rd, 6th, and 12th month after operation was 3.78 ± 3.91, 4.05 ± 4.23, and 4.38 ± 4.93 respectively, and the improvement was not enhanced with increase in time. In addition, 10 patients had WCS score ≥ 8 at the 12th month. Although GIQLI value significantly improved, it was still 103.43 ± 16.42 at the 12th month, showing a difference compared with that of healthy people. The pre-operative APIS did not improve significantly at the 3rd and 6th months after operation, with *p* = 0.61 and *p* = 0.05, respectively. However, APIS significantly improved at the 12th month. Based on postoperative and pre-operative comparisons of the two groups, the surgical approaches of the two groups can improve the clinical symptoms of STC patients, but the observation group was superior to the control group. The results indicate that the preserved length of the ascending colon influences the postoperative outcomes of STC patients treated by LSCACRA. Patients with 2 cm to 3 cm of preserved ascending colon had better postoperative outcomes than patients with 10 cm to 15 cm of preserved ascending colon.

Since this is a single center norandomized historical control study which has some limits, multicenter randomized controlled studies are needed in the future study. At the same time, we will also perform long term follow-up to further evaluate the functional recovery of LSCCRA.

## Conclusions

LSCACRA is an effective approach in the treatment of STC. The reduction of the preserved length of the ascending colon above the ileocecal junction can significantly improve the postoperative outcomes of STC patients treated with LSCACRA. In this study, we recommend that the preserved length of the ascending colon be 2 cm to 3 cm above the ileocecal junction.

## Additional file

Additional file 1:
**Explanation of the reason for carrying on this study.**

## Abbreviations

STC: Slow transit constipation
TC-IRA: Total colectomy with ileorectal anastomosis
SCCRA: Subtotal colectomy with cecorectal anastomosis
SCACRA: Subtotal colectomy with antiperistaltic cecorectal anastomosis
WIS: Wexner incontinence scale
WCS: Wexner constipation scale
GIQLI: Gastro-intestinal quality of life index
LSCACRA: Laparoscopic subtotal colectomy with antiperistaltic cecorectal anastomosis
GITT: Gastrointestinal transit time
ASA: American Society of Anesthesiologists
BM: Bowel movement
SD: Standard deviation
