# Supplementary material for: A prospective comparison of short term results and functional recovery after laparoscopic subtotal colectomy and antiperistaltic cecorectal anastomosis with short colonic reservoir vs. long colonic reservoir
Source: BMC Gastroenterol. 2015 Mar 18;15:30. doi: 10.1186/s12876-015-0257-7 (PMC4367911; doi:10.1186/s12876-015-0257-7)

**Explanation of the reason for carrying on this study**

Since we made the laparoscopic subtotal colectomy with antiperistaltic cecorectal anastomosis , most patients had good recovery, but few of them had signs of abdominal pain and abdominal bloat and had to be re-checked. Six patients among 42 cases did not present significant improvement in abdominal pain and abdominal bloating after the operation, and even reported subjective feelings of enhanced symptoms. They could not endure the pain and had to take long-term treatment in hospitals. They even requested for re-operations.

We made careful examine of these patients and re-evaluate their illness as the following aspects.

1. Constipation was alleviated as shown from the improved WCS index and GIQLI index compared to pre-surgery.
2. The signs of abdominal pain and abdominal bloat were not eased, but aggravated after surgery.
3. No evidence of intestinal obstruction was found.
4. Anastomotic stoma was clear without stenosis which was confirmed by barium enema and colonoscopy.
5. The gastrointestinal transit test (GITT) is normal.
6. No obvious adhesion and malformation of intestine were found in laparoscope.

However, we have found that these patients had the problem of cecum emptying as shown in Fig.1 (GITT after operation: the photos were taken respectively 24, 48 and 72 hours after taking 10 markers at one draught.) and Fig.2 (the picture was taken 72 hours after barium enema.)


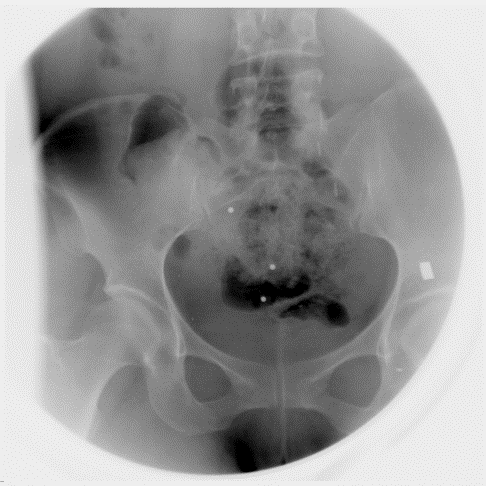


Fig.1-1 In the 24-hour after examine, seven markers were discharged and one was in the caecum and two were in rectum.


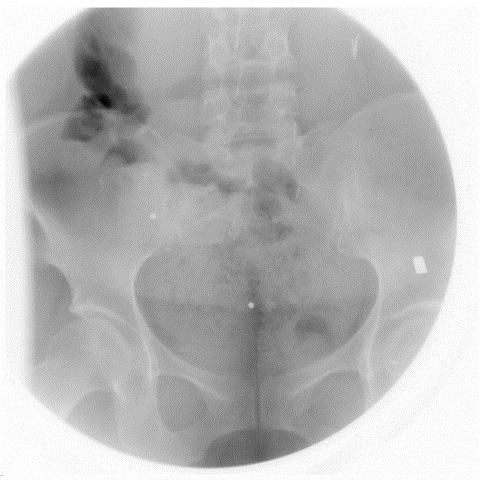


Fig.1-2 In the 48-hour after examine, eight markers were discharged and one was in the caecum and rectum respectively.


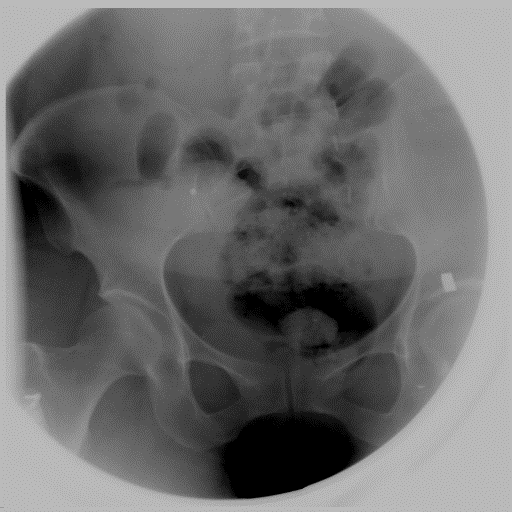


Fig.1-3 In the 72-hour after examine, nine markers were discharged and one was in the caecum.


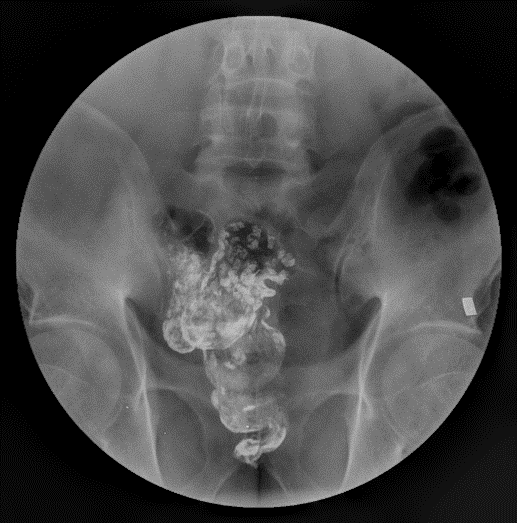


Fig. 2 The barium enema: large amount of barium has dried in block and remained in cecum and colon. Intestine has been emptied and rectum was basically emptied.

We concluded that the residual cecum and colon were shaped as a blind loop-pouch in which the feces moved inversely. Partial feces stayed for a long time in the pouch which was hard to be emptied. The abdominal pain and abdominal bloat was caused by the blind loop-pouch after operation. The pouch was bigger, the illness was more serious.

The reason of abdominal pain and abdominal distention after surgery was explained in the part of Discussion:“This result is attributed to the blind loop formed by the preserved ileocecal junction and the ascending colon. The longer the preserved length, the longer the blind loop. We observed considerable excrement and gas residue in the blind loop because of peristalsis, which formed imposing pressure in the blind loop. The inner pressure in the blind loop may cause abdominal bloating. The large pressure in the cecum leads to reinforced or even spasmodic contraction when the contents of the small intestine enter the colon. Given the excessive length of the blind loop, more intense and longer lasting contractions of the colon will be needed to empty residuals in the blind loop, which generates and worsens colon spasms.”

With the patient’s permission, we decided to conduct another surgery. We had ever thought about cecectomy and ileorectal anastomosis (IRA), but considering patient’s signs of well defecation, improved WCS index and better living quality, the key issue was to solve the abdominal pain and abdominal bloat. If the cecum was cut off, the surgery would possibly result in diarrhea and fecal incontinence. Meanwhile, considering the risk and injury of surgery, recection of cecum has more risk and injury than shortening of cecum. What’s more, if the symptom is not relieved after operation, we also have the chance of recection of cecum.

Based on the theoretical analysis of subtotal colectomy with antiperistaltic cecorectal anastomosis, we concluded that the surgery is significant of retaining ileocecal valve and preventing rotation of blood vessel and intestine in isoperistaltic anastomosis. It is not necessary to keep longer ascending colon and cecum for the function of pouch. In the literature review, there is no final conclusion about what length of ascending colon and cecum should be retained, so theoretically it is feasible to conduct cecum shortening which also observes the surgery principle. Therefore, we at last choose the surgery of cecum shortening. As for the length of shortening, we had careful consideration before the second surgery and thought that the purpose of the new surgery was to solve the problems of feces retention and pneumatosis in the pouch, or minimize even eliminate the pouch. Therefore, we decided to shorten cecum as much as possible and at same time guarantee ileocecal valve and original anastomotic stoma intact. This is the reason why we initially fixed 2-3cm area above the upper edge of ileocecal valve (guaranteeing well function of ileocecal valve and minimized cecum).

The patients recovered very well after the second operation and their illness were obviously relieved in three-month after operation, so we decided to change the operation to shorten the length to 2-3cm above the upper edge of ileocecal valve.

We made operations of cecum shortening on the six patients and achieved favorite results. The detailed materials of the six patients are as follows: the pre-operation and post-operation comparison and analysis of the effects in two surgeries.
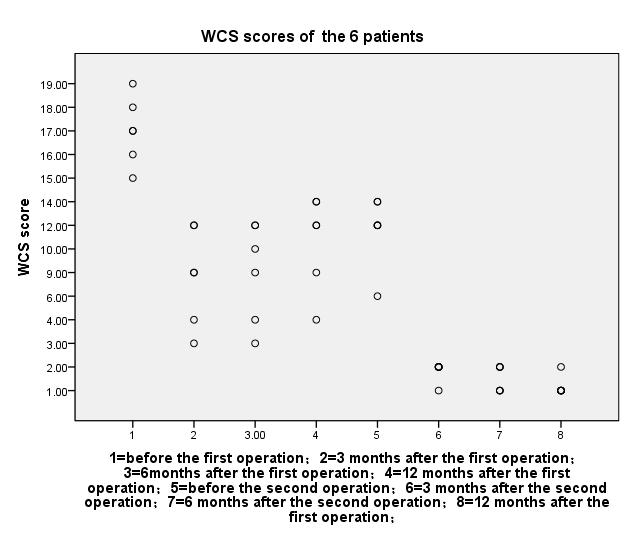

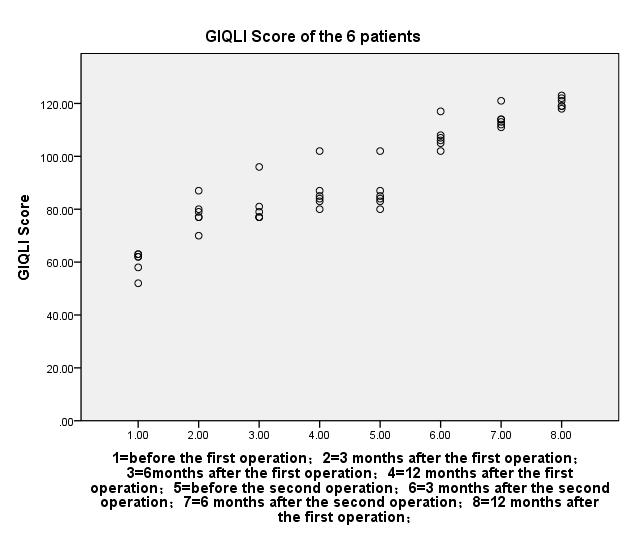

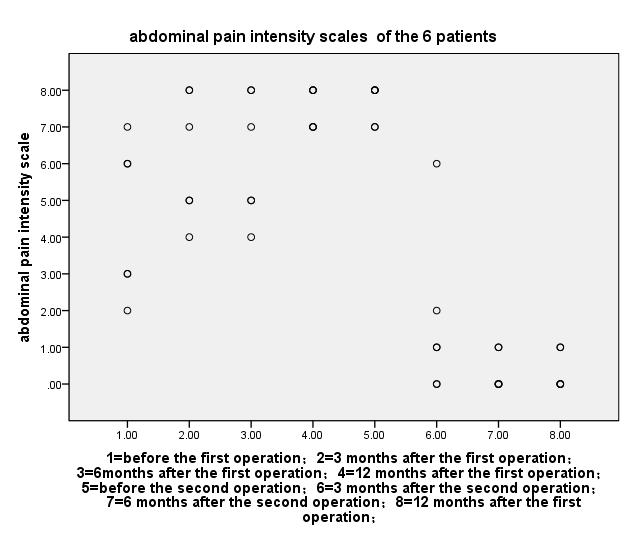

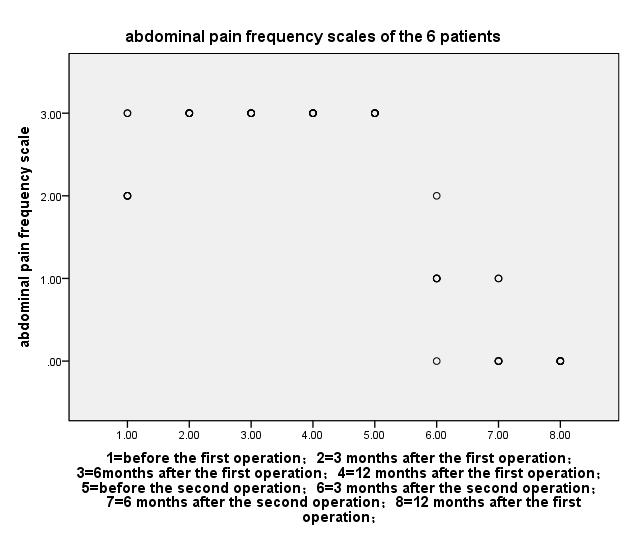


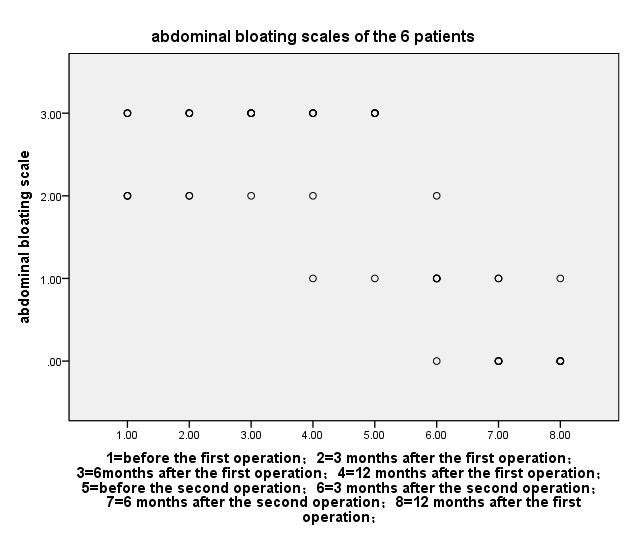

Supplement: Additional file 1: — Explanation of the reason for carrying on this study. [file 12876_2015_257_MOESM1_ESM.docx]
